# Traceless Regulation of Genetic Circuitry

**DOI:** 10.1002/advs.202519848

**Published:** 2025-12-27

**Authors:** Gokberk Unal, Martin Fussenegger

**Affiliations:** ^1^ Department of Biosystems Science and Engineering ETH Zurich Basel Switzerland; ^2^ Faculty of Science University of Basel Basel Switzerland

**Keywords:** cell circuit, gene switch, synthetic biology, traceless

## Abstract

The advent of synthetic biology, enabling the construction of synthetic genetic circuitry with designed functionality, has has a revolutionary impact on medicine, agriculture, sustainable energy, and the industrial production of high‐value compounds over the last few decades. Gene switches have an indispensable role as regulators of such systems. Despite the early introduction of chemically inducible switches to regulate genetic circuitry, ‘traceless’ physical cues (e.g., light, heat, sound, magnetism, electricity, and mechanical force) can provide greater specificity, higher spatiotemporal resolution, more flexible switching patterns, and better compatibility with bioelectronic interfaces, which is of particular significance given the rise of electrogenetics. Indeed, traceless gene switches are on a path to become universal biological control ports interfacing physiology with the electronic world. In this review, we discuss the impact, challenges, and prospects of physically inducible, traceless gene switches in the context of recent cutting‐edge applications.

## Graphical abstract

Energy‐based, as opposed to molecular, control offers unprecedented improvements in key circuit parameters. This review summarizes the fundamentals of such traceless switches, categorizes them by trigger modalities, and compares and contrasts distinct advantages as well as shortcomings of each kind. Subsequent sections highlight current translational barriers and outline prospective applications across different disciplines.

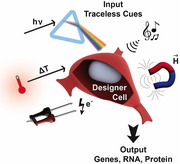

## 1 Introduction

All organisms, ranging from humans and other primates to the most primitive archaic bacteria, possess intricate, intertwined control modules that autonomously regulate protein, RNA, and DNA expression at the cellular level [1, 2, 3]. The advent of synthetic biology has led to increasing interest in the identification of minimal biological building blocks that might be repurposed to construct new functionalities [4, 5], and early work on the lac operon introduced the idea of a genetic circuit with logic‐like control, presenting DNA as a programmable entity for the first time [6, 7]. The subsequent emergence of recombinant DNA technology made possible the cutting and pasting of genomic sequences using restriction endonucleases and ligases, which ultimately led to the creation of synthetic gene circuits in the early 2000s, marking the birth of synthetic biology as a distinct discipline [4, 8, 9]. Since then, synthetic biology has had a dramatic impact in the fields of medicine, agriculture, sustainable energy, and manufacturing [10, 11, 12, 13, 14, 15, 16, 17, 18, 19, 20, 21, 22, 23, 24, 25].

Gene switches constitute an indispensable component for the regulation of synthetic genetic circuitry [26, 27, 28], enabling systems to be turned on and off as required. Furthermore, the incorporation of multiple switch elements in higher‐tier programmable logic gates can add additional layers of control and thereby enhance safety [29, 30]. Numerous gene switches have been developed by synthetic biologists within the last two decades, affording a toolbox of natural (transcription factors, receptors, channels) and synthetic modules with a broad range of chemical triggers, including proteins, short peptides, hormones, antibiotics, vitamins, food additives, cosmetics, and inhalable substances [31, 32, 33, 34, 35, 36, 37, 38]. Gene switches with chemical triggers undoubtedly offer interesting opportunities for clinical applications, with diverse possibilities for administration of trigger compounds (percutaneous, oral, pulmonary, intravenous, ophthalmic) [27].

Nevertheless, chemical trigger compounds have a number of important limitations [39]. For instance, depending on the parameters of the switch sensor and the desired timeline of the switch output, the pharmacokinetic properties may be restrictive [26, 40]. Specifically, water‐soluble vitamins like thiamine and niacin are cleared within hours, whereas fragment crystallizable (Fc) tagged peptides may have half‐lives that span weeks [41, 42, 43]. This also raises the issue of reversibility in vivo, as once the trigger compound is administered, the only options to turn off a chemical switch are to wait until the trigger compound is eliminated or to introduce a secondary, ‘kill’ switch that short‐circuits the primary one [44, 45, 46, 47, 48]. Furthermore, regardless of the initial method of administration, most chemical trigger compounds eventually enter the circulation [49], so there is a potential risk of toxicity. The possibility of off‐target activation must also be considered [50]. Furthermore, even if chemical sensors are synthetically engineered to respond to a specific inducer, they may detect structural analogs, albeit at varying sensitivities, leading to unintentional circuit activation even in the absence of the actual trigger compound. Thus, despite the advantages that chemical switches offer, limitations of spatiotemporal precision and ligand exclusivity still pose challenges. Therefore, there has been increasing interest in developing ‘traceless’ gene switches employing physical stimuli [51].

In this review, we discuss the fundamentals of such traceless switches, categorize traceless gene switches by trigger modalities, and compare and contrast distinct advantages as well as shortcomings of each kind. Subsequent sections highlight current translational barriers and outline prospective applications across medicine, industry, and agriculture.

## 2 Physically Triggered Gene Switches

**FIGURE 1:**
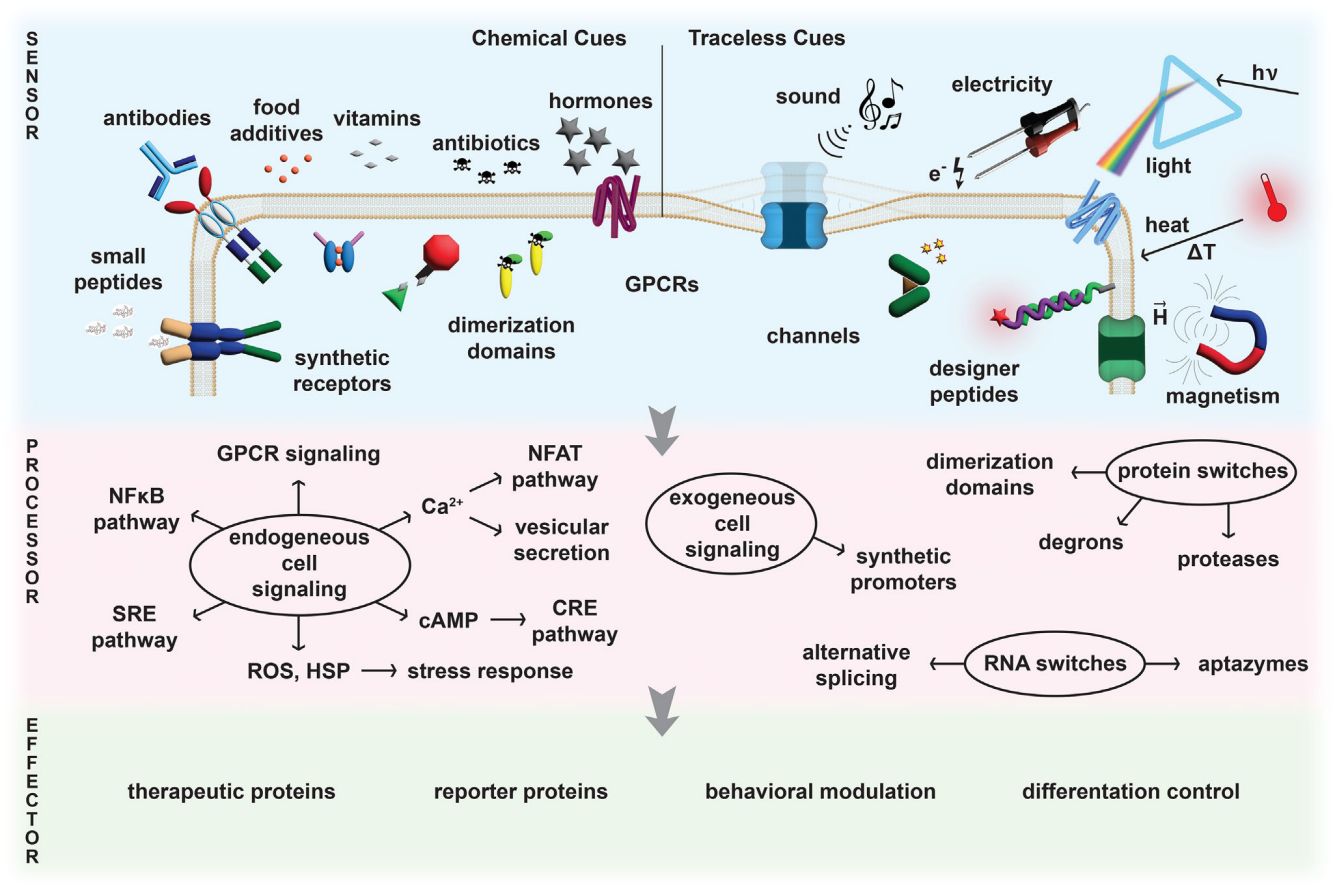
Gene switch blueprint. Genetic circuits have distinct sensor, processor, and effector modules. Gene switches are categorized depending on the nature of the inducer. Chemical induction can be triggered by small molecules such as antibiotics, vitamins, food additives, inhalable substances, cosmetics, short peptides, and hormones, or larger molecules like antibodies. Conversely, traceless switches are trigger‐inducible by various forms of energy such as light, sound, electricity, magnetism, or heat. Trigger‐sensing is achieved through various tools such as G‐protein coupled receptors (GPCRs), synthetic receptors, ion channels, dimerization domains, and designer peptides. The input signal is then cellularly processed through either endogenous signaling pathways (e.g., Ca^2+^‐dependent, nuclear factor of activated T cells (NFAT) pathway, cyclic adenosine monophosphate (cAMP)‐dependent cAMP response element (CRE) pathway, sterol response element (SRE) pathway, nuclear factor kappa light chain enhancer of activated B cells (NFκB) signaling, reactive oxygen species (ROS)/heat shock protein (HSP)‐dependent stress response), and exogenous signaling pathways involving synthetic promoters, or protein/RNA switches. Ultimately, the processed input signal is rewired to the desired output, which, depending on the application, can be expression of therapeutic or reporter proteins, cell behavioral modulation, or differentiation control.

Various naturally occurring proteins respond to specific forms of energy [52, 53, 54]. For example, ocular receptors initiate downstream cell signaling cascades upon exposure to distinct wavelengths of light, while heat‐shock factors trigger the transcription of genes regulated by heat shock elements upon sensing a change in temperature, and mechanoreceptors are activated by certain frequencies of soundwaves [55, 56, 57]. These natural interactions can be leveraged to trigger ‘traceless’ gene switches, and are especially advantageous in cases where spatiotemporal resolution is critical (Figure 1) [26]. The ability to control biological systems with energy instead of chemical triggers can provide millimetric spatial precision, free from environmental influences [51]. Thus, the use of physical cues not only eliminates the unavoidable consequence of systemic exposure to chemical inducers, but also offers higher temporal resolution and the ability to trigger complex induction patterns that go beyond simple on/off behavior, providing dimmer switch‐like control [51]. Furthermore, since physical triggers are generated in situ via electrical devices, they open up new dimensions of compatibility with bioelectronic interfaces, which is of particular significance given the rise of electrogenetics [58, 59, 60, 61]. In the following sections we review the different modalities available for physical triggering of gene switches.

### 2.1 Optogenetic Switches

**FIGURE 2:**
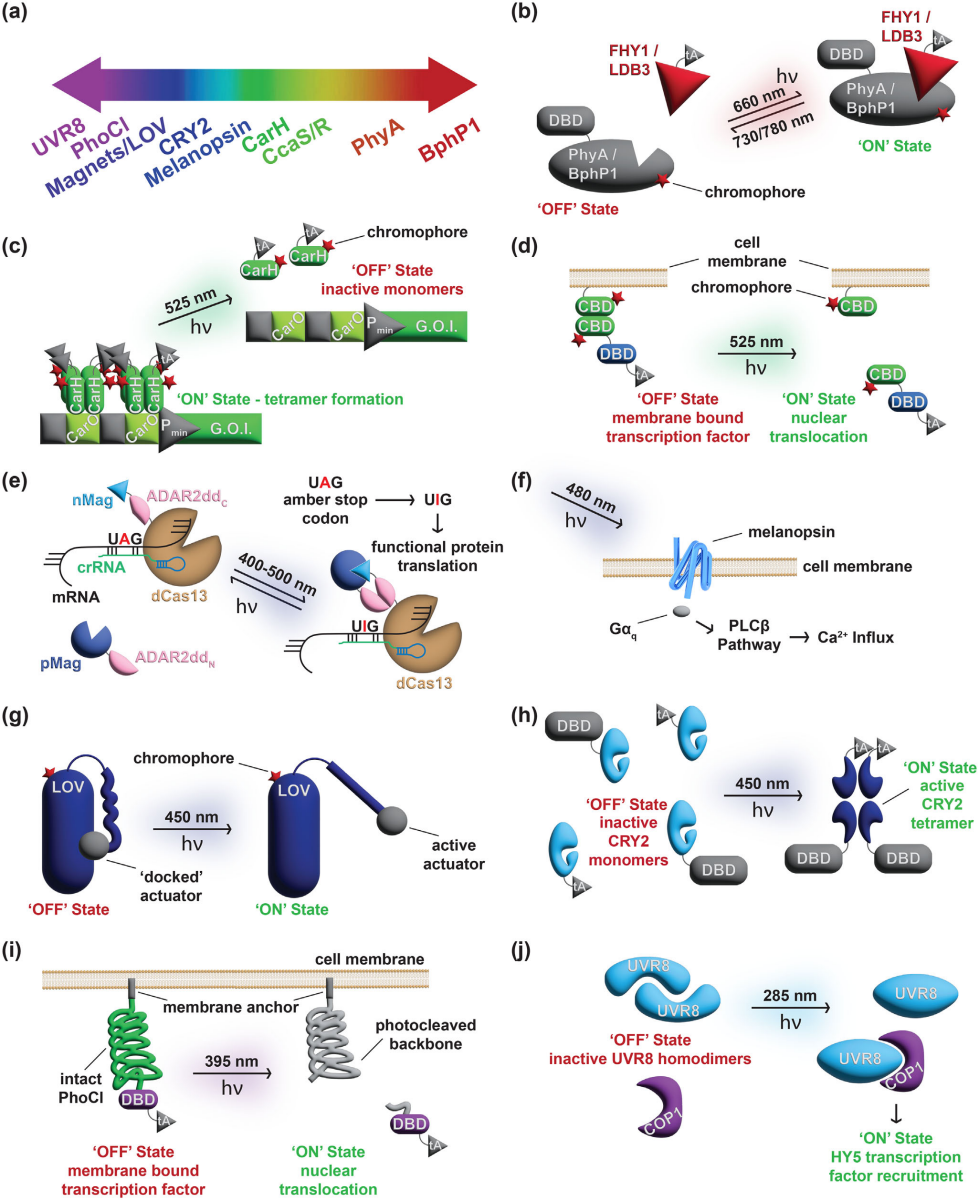
Optogenetic switches. (a) Various natural and synthetic proteins are capable of sensing distinct wavelengths of light. (b) The REDMAP system utilizes heterodimers of phytochrome A (PhyA)/far‐red elongated hypocotyl1 (FHY1) of plant origin that reversibly dimerize upon exposure to red light at 660 nm. By synthetically fusing one heterodimer to a DNA binding domain (DBD) and the other to a transactivator (tA), transcription of any given gene of interest is initiated on demand by induction with red light. The REDLIP system utilizes the same design as the REDMAP system but employs heterodimers of bacteriophytochrome photoreceptor 1 (BphP1)/LIM domain binding 3 (LDB3) of bacterial origin, with a slightly increased monomerization wavelength. (c) The bacterial transcription factor CarH is synthetically fused to a transactivator and is constitutively expressed. By default, the fusion protein forms tetramers and binds its cognate DNA sequence CarO in the nucleus to initiate gene expression. Green light exposure at 525 nm monomerizes the fusion protein, leading to its dissociation from the expression cassette, yielding an optogenetic OFF‐switch. (d) Conversely, the Glow Control system utilizes the same interaction with a different design to yield an ON‐switch where two distinct fusion proteins containing the cobalamin binding domain (CBD) of CarH are used. One homodimer is tethered to the inside of the cell membrane with a myristoylation domain, while the second homodimer is fused to a synthetic transcription factor, which is only released upon induction with green light. (e) Upon blue light exposure, nMag and pMag dimerize, leading to the assembly of split adenosine deaminase fragments (ADAR2ddN and ADAR2dd_C_). This in turn allows targeted base editing, converting the default amber stop codon UAG into UIG, achieving translation of a functional transgene. (f) The G protein‐coupled receptor melanopsin senses blue light at approximately 480 nm to trigger the phospholipase C beta (PLCβ) signaling pathway, ultimately leading to Ca^2+^ influx, which can be used to initiate transgene expression through a synthetic NFAT promoter. (g) The blue‐light‐responsive LOV domain docks the C‐terminally fused actuator by default, rendering it inactive. Upon photostimulation at 450 nm, the Jα domain unfolds, revealing the actuator. (h) The cryptochrome (CRY) protein exists by default in its inactive form, but undergoes conformational change upon induction with blue light, leading it to tetramerize. When two CRY‐based fusion proteins are present, one fused to a DNA binding domain and the other to a transactivator, the system operates as a blue‐light‐inducible gene switch. (i) The photocleavable protein PhoCl undergoes spontaneous dissociation upon exposure to violet/UV‐A light to release a short C‐terminal peptide. Tethering PhoCl with an N‐terminal myristoylation domain and fusing a transcription factor at the C‐terminus affords a violet‐light‐inducible photoswitch. (j) UVR8 forms homodimers by default and monomerizes upon exposure to UV‐B. The active UVR8 monomers can then interact with the COP1 protein to activate its cognate signaling cascade.

The use of light as a trigger for gene expression has many advantages [62]. Spectral multiplexing of orthogonal light sensors and bioluminescent proteins can achieve synthetic communication between designer cells [63]. Furthermore, optogenetic switches have the highest spatiotemporal precision of all physically triggered gene switches [64]. Indeed, focused beam lasers can target areas as small as 7.5 µm^2^, which is enough to induce single cells with a temporal resolution of millisecond order [65]. Consequently, numerous optogenetic switches have been developed capitalizing on both natural and synthetic light sensors (Figure 2a). Longer wavelengths of light such as red (640–700 nm), far‐red (700–800 nm), and near‐infrared (NIR, 800–2500 nm) offer greater tissue penetration with low phototoxicity, making them suitable for deep‐tissue applications such as brain stimulation, gene expression modulation, cancer therapy, and metabolic disease treatments [66]. The REDMAP and REDLIP systems use a heterodimer of phytochrome A (PhyA)/far‐red elongated hypocotyl1 (FHY1) of plant origin and a heterodimer of bacteriophytochrome photoreceptor 1 (BphP1)/LIM domain binding 3 (LDB3) of bacterial origin, respectively, to yield highly sensitive xenogeneic photoswitches (Figure 2b) [67, 68]. Moving down in wavelength through the visible light spectrum, the bacterial transcription factor CarH and its cognate DNA binding domain CarO have been combined to achieve optical control of gene expression using green light (∼525 nm), affording 350‐fold inducibility at the cost of reduced tissue penetration (Figure 2c) [69]. Similarly, the Glow Control system also utilizes the green‐light inducible dimerization of the cobalamin binding domain (CBD) of CarH to express and release transgenic insulin, enabling effective treatment of experimental diabetes controlled by an Apple watch (Figure 2d) [70]. Shorter wavelength‐triggered systems such as blue‐light‐activated melanopsin receptor signaling, light‐oxygen‐voltage (LOV) domain conformational change, cryptochrome 2 (CRY2) homodimerization, negative and positive Magnet (nMag and pMag) heterodimerization, etc., have shorter induction times and require less power, but suffer from poor tissue penetration and relatively higher phototoxicity (Figure 2e–g) [66, 71, 72, 73]. The recently engineered photocleavable protein (PhoCl) introduced a new optogenetic induction mechanism whereby the sensor protein undergoes spontaneous dissociation upon exposure to light in the wavelength range between ultraviolet (UV) and blue (Figure 2h) [74, 75]. Lastly, the plant photoreceptor UVR8 monomerizes under illumination with UV‐B (280–320 nm) and interacts with the Constitutive Photomorphogenic 1 (COP1) protein, activating its cognate signaling cascade (Figure 2i) [76]. Unfortunately, most, though not all, optogenetic systems require exogenous chromophores and thus cannot be considered truly traceless circuits. Nevertheless, thanks to their inherently high spatiotemporal precision and multiplexing capabilities at mutually exclusive wavelengths, optogenetic switches have significant appeal for a variety of applications.

### 2.2 Thermogenetic Switches

**FIGURE 3:**
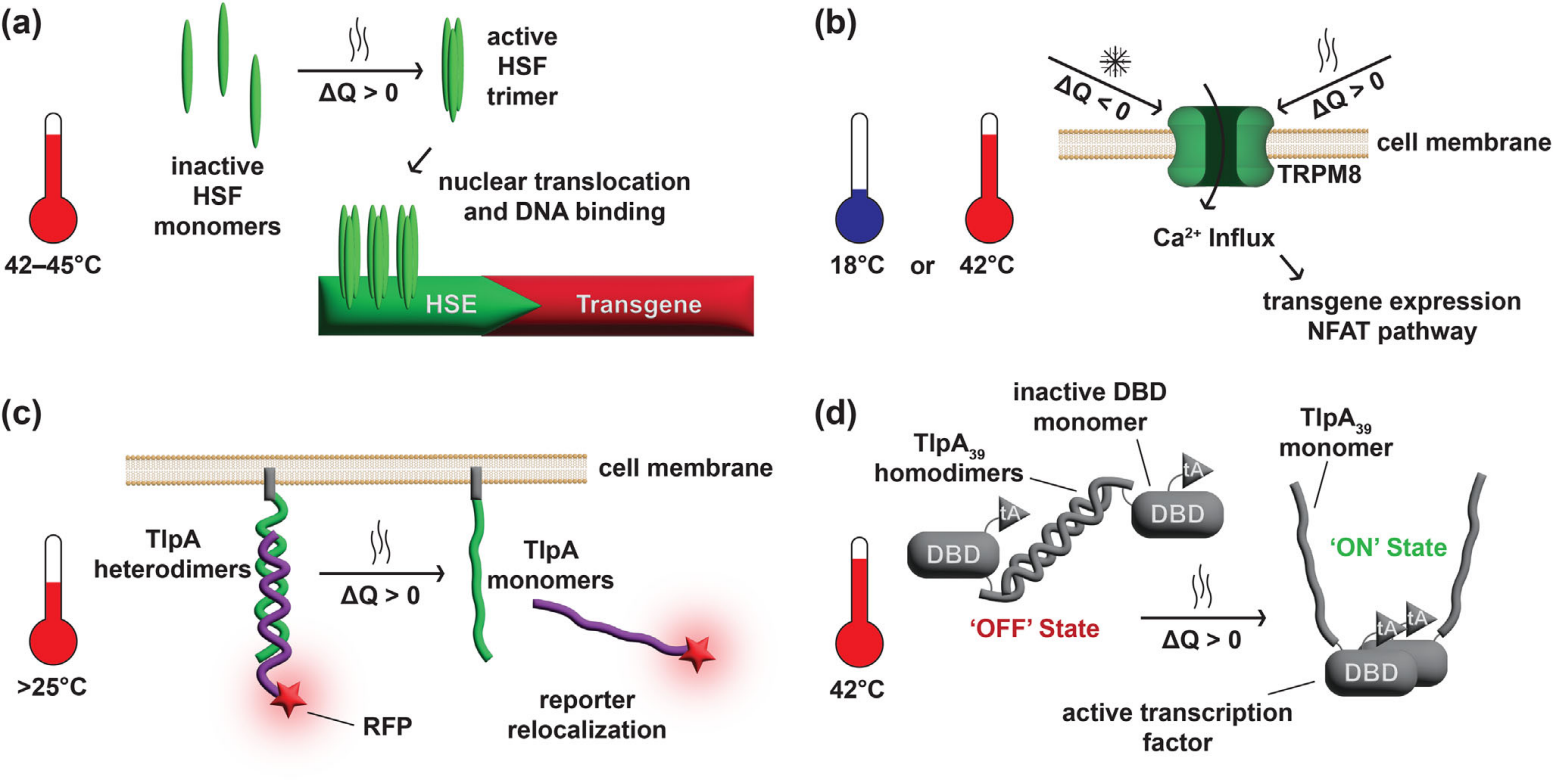
Thermogenetic switches. (a) The endogenous heat shock response involves heat shock factors (HSFs) that are present in their inactive monomeric form at homeostatic temperatures. When the cell temperature rises above 42°C, HSFs form trimers, translocate to the nucleus, and bind to their cognate heat shock element (HSE) sequences. By incorporating an HSE‐driven synthetic expression cassette, the endogenous heat shock response is utilized to achieve transgene expression on demand by heat induction. (b) The transient receptor potential cation channel subfamily M member 8 (TRPM8) is closed at homeostatic temperature. When the temperature goes below 18°C or above 42°C, the channel opens to allow Ca^2+^ influx, activating the Ca^2+^‐dependent NFAT pathway. (c) TlpA heteromers exist as dimers at room temperature and begin to undergo conformational change upon heating with a gradually increasing rate of dissociation as the temperature increases up to 45°C. By tethering one heteromer to the cell membrane through a myristoylation domain and fusing an actuator to the second one, a heat‐inducible switch is obtained. (d) Similarly, the TlpA mutant TlpA_39_ forms homodimers at temperatures at or below homeostatic temperature. Upon heating to 42°C, the homodimers dissociate, allowing the transcription factors to interact and activate transgene expression.

Heat has recently been introduced as a trigger for transgene expression in synthetic circuits [77]. Although the heat shock elements (HSE) endogenous to mammalian cells were identified in the 1980s, engineered thermogenetic synthetic circuits have only risen to prominence within the last decade [78]. Importantly, the endogenous nature of the thermogenetic circuit components greatly reduces the risk of immunogenicity, which is a major point of consideration when engineering designer circuits [79]. While heat shock elements intrinsically control the cellular stress response to variations from homeostatic temperature, it is possible to hijack the wild‐type machinery and use it to control transgene expression [79]. Heat shock factor 1 (HSF1) is a predominantly monomeric, cytoplasmic protein at homeostatic temperature (∼37°C). At elevated temperatures associated with high‐grade fever and above (>40°C), HSF1 undergoes conformational changes and forms homotrimers that can translocate to the nucleus, bind to their cognate HSE, and promote gene expression (Figure 3a) [80]. Endogenous HSE‐regulated genes encode heat shock proteins (HSPs) that protect cellular function at elevated temperatures by ensuring that functional proteins are not misfolded, regulating apoptosis, or activating immune response. A synthetic expression cassette driving HSE‐regulated transgene expression can take advantage of this machinery, providing a simple yet effective thermogenetic circuit. The Cool_Sens_ thermogenetic switch utilizes an endogenous heat responsive ion channel, transient receptor potential cation channel subfamily M member 8 (TRPM8) (Figure 3b) [81]. TRPM8, also known as the cold and menthol receptor 1 (CRM1), allows Na^+^ and Ca^2+^ entry into the cell, leading to membrane depolarization and subsequent activation of Ca^2+^‐mediated signaling cascades upon sensing a decrease in temperature (<20°C) or a menthol‐induced pseudo‐cooling effect. Interestingly, TRPM8 can also initiate membrane depolarization at elevated temperatures (>42°C). TlpA, a heat‐sensitive protein of bacterial origin, acts as a thermosensor over a wide range of temperatures (25°C–45°C). The coiled‐coil domain gradually unfolds as the temperature increases, leading to dissociation of the dimers. This interaction can be used for thermal control of protein dimerization (Figure 3c) [78]. More recently, the human enhanced gene activation thermometer (HEAT) utilized a mutant variant TlpA_39_ to control transgene expression via heat transfer to treat experimental type‐1 diabetes in mice (Figure 3d) [82]. The major difficulty thermogenetic circuits face is temperature homeostasis. While it is possible to induce cells in vitro by exposing them to non‐physiological temperatures, subcutaneous or intraperitoneal cell implants containing the thermogenetic circuits cannot be induced as efficiently in vivo [81, 82].

### 2.3 Sonogenetic Switches

**FIGURE 4:**
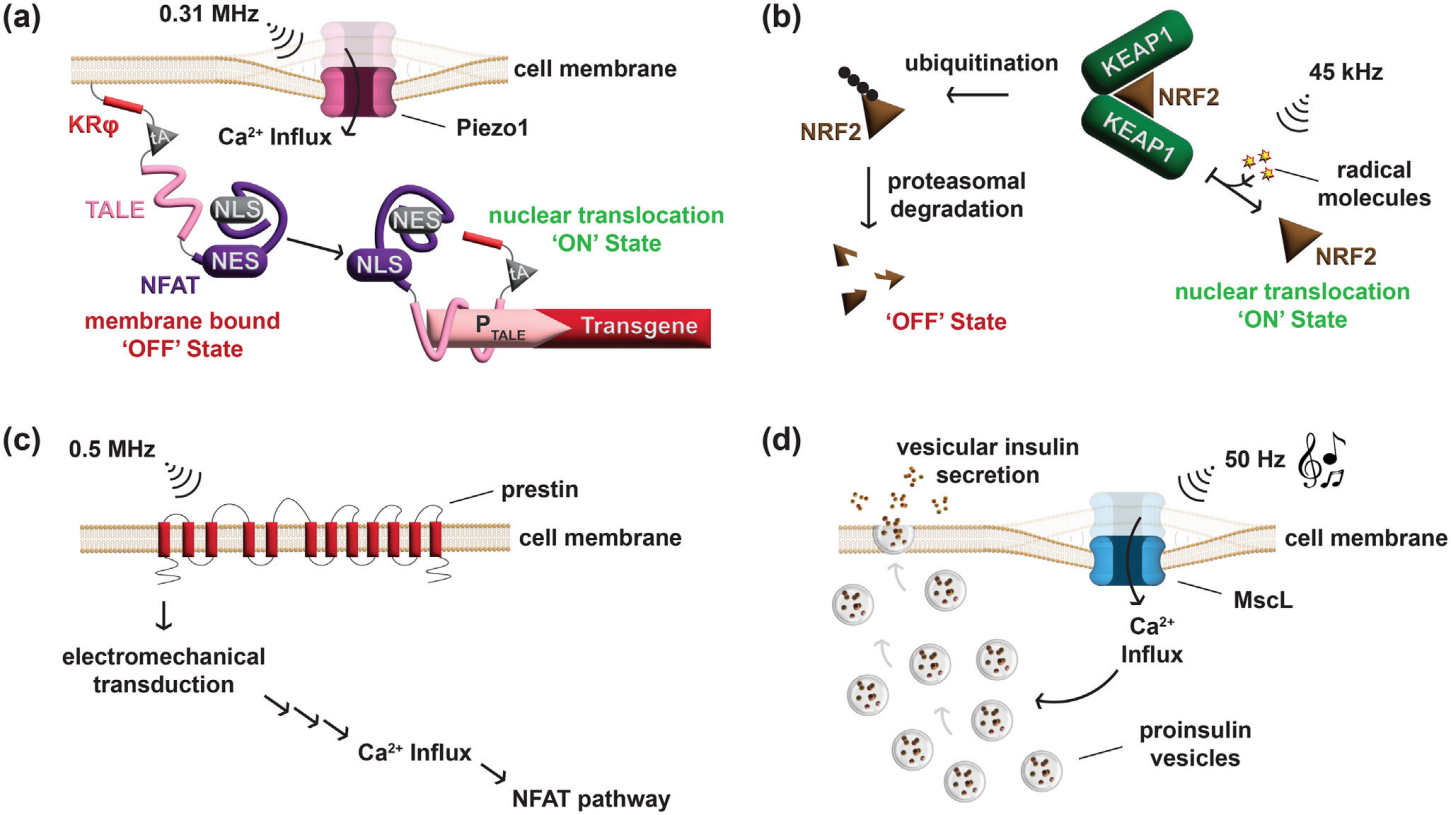
Sonogenetic switches. (a) Piezo1 allows the influx of Ca^2+^ ions when placed under mechanical stress due to ultrasound stimulation, leading to the partitioning of the KRφ peptide in an ionic strength‐dependent manner. The Ca^2+^‐responsive transcription factors (CaTFs) then translocate to the nucleus to drive transgene expression from a synthetic reporter construct containing ten repeats of the transcriptional‐activator‐like effector (TALE) binding site. (b) The nuclear factor erythroid‐2 related factor 2 (NRF2) is ubiquitinated by Kelch‐like ECH‐associated protein 1 (KEAP1) and subsequently subjected to proteasomal degradation. Brief stimulation with low‐frequency ultrasound initiates the intracellular formation of non‐toxic levels of reactive oxygen species (ROS), which prevent the ubiquitination of NRF2, allowing downstream signaling to occur. (c) Prestin is a large membrane protein with 12 transmembrane domains and upon stimulation with low‐frequency ultrasound, it initiates electromechanical transduction that ultimately leads to the influx of Ca^2+^ ions, activating the Ca^2+^‐dependent NFAT pathway. (d) Auditory stimulation with music causes membrane deformation in designer cells overexpressing the mechanosensitive ion channel, MscL, leading to the influx of Ca^2+^ ions. The increase in cytoplasmic Ca^2+^ concentration initiates the secretion of proinsulin vesicles through a beta cell‐mimetic mechanism.

Compared to most other traceless genetic circuits that require a specialized, non‐everyday device to trigger induction, sonogenetic switches offer greater practicality and user‐friendliness [83, 84]. The deep‐tissue penetration of soundwaves enables non‐invasive access to internal organs with spatial control at a millimeter‐scale, with minimal or no side effects. Depending on the induction parameters such as frequency, amplitude, and exposure time, soundwaves can trigger genetic circuits via different mechanisms. For instance, prolonged exposure to higher frequencies of sound (e.g., ultrasound) can cause localized elevations in temperature, which can be utilized as a trigger for sonogenetic switches [85]. However, since these overlap with thermogenetic switches, this section deals exclusively with sonogenetic switches that operate through electromechanical transduction. Piezo1 is a highly conserved mechanosensitive ion channel that is present in many species including humans. Upon activation by mechanical stress such as membrane stretching, tissue compression, or shear flow, Piezo1 allows the influx of cations. Low‐frequency ultrasound stimulation for 2 h provides sufficient mechanical force to trigger Piezo1 and subsequently activate the NFAT‐based signaling pathway through Ca^2+^ influx (Figure 4a) [85]. Ultrasound stimulation also causes the intracellular formation of non‐toxic levels of reactive oxygen species (ROS). Indeed, 40‐s low‐frequency ultrasound exposure promotes dissociation of the nuclear factor erythroid‐2 related factor 2 (NRF2) from Kelch‐like ECH‐associated protein 1 (KEAP1), preventing its ubiquitination and subsequent proteasomal degradation. Free NRF2 is then able to translocate into the nucleus, where it binds to antioxidant response element binding sites to drive gene expression, yielding a sonogenetic switch (Figure 4b) [86]. Prestin, an auditory‐sensing motor protein endogenously found in the outer hair cells of the inner ear of mammalian cochlea, confers low‐frequency ultrasound sensitivity by altering membrane mechanical properties, lowering the activation threshold of various mechanosensitive channels (Figure 4c) [87]. Almost all sonogenetic switches developed within the last decade use ultrasound as trigger. However, the recently engineered music‐inducible cellular control (MUSIC) system is the first audible‐sound‐inducible genetic circuit. Leveraging the large‐conductance mechanosensitive channel (MscL) of bacterial origin as well as the beta cell‐like vesicular insulin secretion mechanism, MUSIC_INS_ cells were able to treat experimental type 1 diabetes upon triggering by exposure to various genres of music, with each song producing a distinct secretion profile (Figure 4d) [84]. However, minimizing off‐target activation is a key aspiration in designing gene switches, and circuits that are triggered by audible sound or low‐frequency ultrasound with short induction times and fast release mechanisms risk off‐target activation, as an average person is involuntarily exposed to such sounds on a daily basis [88].

### 2.4 Mechanogenetic Switches

Besides sonogenetic switches that achieve electromechanical transduction through ultrasound and audible sound triggers, purely mechanogenetic circuits are emerging as a distinct modality [89, 90]. Mechanoswitches rely directly on mechanical forces (compression, tension, or shear) to activate signaling pathways, utilizing various mechanosensitive elements including receptors, channels, and promoters [91]. A recently developed mechanoswitch in chondrocytes drives an anti‐inflammatory response to mechanical stress, offering autonomous control of arthritis therapy [54]. Such circuits have enhanced translational potential, as they broaden the concept of traceless gene switches by exploiting everyday biomechanical inputs that do not require specialized devices.

### 2.5 Electrogenetic Switches

**FIGURE 5:**
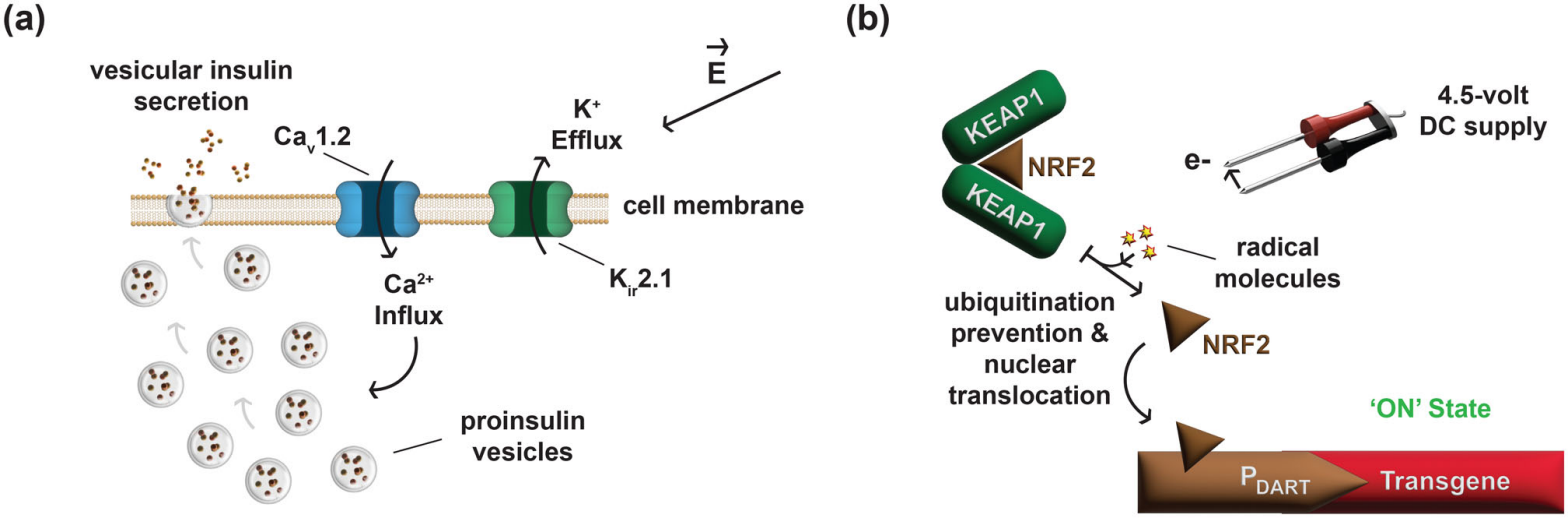
Electrogenetic switches. (a) The synthetic overexpression of the L‐type voltage‐gated calcium channel Ca_v_1.2 and the inwardly rectifying potassium channel K_ir_2.1 endows beta cells with electrosensing capabilities. Upon exposure to an extracorporeally generated electric field, the sequential efflux of K^+^ and influx of Ca^2+^ ions activate the endogenous vesicular secretion mechanism of beta cells. (b) Electrostimulation with direct current leads to the formation of ROS, which prevents the ubiquitination and subsequent proteasomal degradation of NRF2, allowing it to translocate to the nucleus and initiate transgene expression from a synthetic reporter construct (P_DART_) containing various repeats of antioxidant‐response element (ARE) operator sites.

Many cells possess an intrinsic capacity to interpret electrical signals for the regulation of cellular processes such as growth and repair functions [92]. Electrically active proteins such as ion channels, electrogenic pumps, gap junction and synapse proteins, and ionotropic and metabotropic receptors offer many opportunities for the engineering of electrogenetic gene switches [93, 94, 95, 96]. A key advantage of using electrical energy as opposed to other traceless physical cues is the elimination of a mediating transducer. Indeed, a significant portion, though not all, of physical gene switches function through membrane potential‐related machinery and subsequent Ca^2+^‐dependent signaling [51]. Direct actuation through electrical signals can therefore not only simplify the genetic circuitry, but also prevent energy dissipation in surrounding tissues [51]. The Electro_β_ system utilizes the L‐type voltage‐gated calcium channel Ca_v_1.2 and the inwardly rectifying potassium channel K_ir_2.1 together with the endogenous vesicular secretion mechanism of beta cells to secrete therapeutic insulin on demand, upon wireless stimulation with an extracorporeally generated electric field (Figure 5a) [97]. The direct current‐actuated regulation technology (DART) uses acupuncture needles as electrodes to electrostimulate subcutaneous designer cells with direct current from standard AA batteries [98]. A stimulation as short as 20 s at 4.5 V generates sufficient levels of ROS to activate the KEAP1/NRF2 stress response and induce expression of therapeutic amounts of insulin from engineered cells in vivo (Figure 5b). The importance of electrogenetic switches arguably goes further than that of chemical or other traceless switches, as electrogenetics can seamlessly interface biological systems with digital electronic counterparts [58, 60, 61]. Indeed, in addition to the switch modalities, ultra‐sensitive sensors and a range of power generators have been developed. For example, the versatile bioelectronic interface (VIBE) is able to detect sub‐nanomolar concentrations of blood biomarkers through changes in the impedance of designer cells seeded on interdigital electrode chips [99]. Additionally, a recently developed metabolic fuel cell system can harvest electrical energy from normal metabolic activities to sustainably power coupled electrogenetic circuits [100]. The moisture‐driven electrical power generator (MODEG) uses the water vapor in exhaled breath while the piezoelectric power generator uses a coin‐sized finger‐push implant to generate 450–600 mV and 2 V output voltage, respectively [101, 102]. With the advent of wearable electronics and bioelectronic implants, electrogenetic gene switches have the potential to form the conduit between biology and electronics. One prominent challenge facing electrogenetics stems from incompatibility with the aquatic environment that is the human body [103, 104, 105]. While other types of energy (light, sound, heat, etc.) can seamlessly induce cells, non‐wireless electrical induction is prone to short circuits [106]. Especially in cases where uninterrupted functionality is critical, preventing such issues is of utmost importance [107].

### 2.6 Magnetogenetic Switches

**FIGURE 6:**
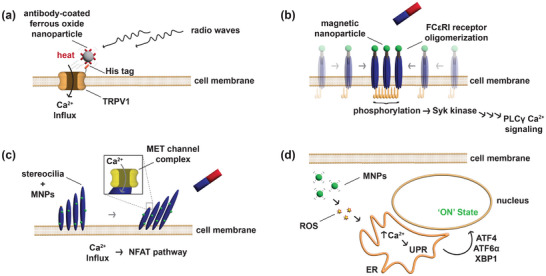
Magnetogenetic switches. (a) Antibody‐coated ferrous oxide nanoparticles bind to His‐tagged TRPV1 channels. Upon stimulation with radio waves, the nanoparticles emit heat to their surroundings due to magnetic hyperthermia, leading to magneto‐thermal induction of the TRPV1 channel and subsequent Ca^2+^ influx, which may be leveraged to activate transgene expression with a synthetic, Ca^2+^‐responsive reporter construct. (b) MNP‐bound FcεRIs are uniformly distributed on the cell membrane by default. In proximity to a magnetic probe, the magnetic pull on the MNPs leads to clustering and oligomerization of the FcεRIs, activating the endogenous signaling pathway. (c) MNPs are coated with silica, polyethylene glycol (PEG), and concanavalin A (conA) that binds to glycoproteins abundant on the hair bundle surface. In proximity to a magnetic probe, the MNP‐bound stereocilia are pulled in the direction of the magnetic field, leading to the opening of the MET channel complex and subsequent Ca^2+^ influx. (d) Magnetic stimulation of cytosolic MNPs leads to the formation of ROS in neurons. The subsequent polarization of the ER membrane causes Ca^2+^ retention, which leads to the release of various anti‐stress transcription factors such as activating transcription factors 4 and 6α (ATF4, ATF6α) as well as the X‐box binding protein 1 (XBP1) into the nucleus.

Magnetic fields can also be utilized to trigger traceless gene switches with sub‐micrometer and millisecond spatiotemporal resolution [108]. Depending on their working principle, such switches are further subcategorized as magneto‐thermal, magneto‐mechanical, or magneto‐biochemical [109]. Magneto‐thermal switches achieve heat activation of either TRP ion channels or the cellular heat shock response through radio frequency (RF, 400 kHz–40 MHz, 1–30 mT) stimulation of magnetic nanoparticles (MNPs) (Figure 6a) [110, 111]. The nanoparticle size and the magnetic material, as well as the amplitude and the frequency of the RF field, are adjustable parameters that influence the amount of heat generated on demand through magnetic hyperthermia. Magneto‐biochemical switches leverage the fluidity of the mammalian cell membrane to cluster otherwise sparsely distributed, MNP‐bound membrane proteins using a magnetic probe (Figure 6b) [112]. The subsequent oligomerization of the high‐affinity (Immunoglobulin E (IgE) receptor (FcεRI) initiates downstream Ca^2+^ signaling. Magneto‐mechanical induction involves the conversion of a mechanical stimulus into an electrical or biochemical signal. This can be achieved through the coupling of MNPs with stereocilia of inner ear hair cells and subsequent activation of the mechanoelectrical transduction (MET) channel, allowing Ca^2+^ influx (Figure 6c) [113]. Other examples of magneto‐mechanical stimulation involve the manipulation of individual Notch receptors of E‐cadherin at the cell surface through magnetic push/pull using a magnetic probe. Additionally, magnetic stimulation of intracellular MNPs can cause the formation of ROS in neurons, followed by Ca^2+^ retention in the ER, unfolded protein response (UPR), and subsequent translocation of various anti‐stress transcription factors into the nucleus (Figure 6d) [114, 115]. Unfortunately, the dependency on exogenous MNPs seriously limits the practicality of magnetogenetic switches. Indeed, the introduction of nanoparticles in a cellular implant can not only cause an immune response and cytotoxicity, but may also reduce longevity [116, 117]. Moreover, the dependency on exogenous MNPs arguably implies that magnetogenetic switches are not truly traceless [118].

Overall, these unique, traceless modalities showcase how energy‐based, as opposed to molecular, control can enable unprecedented improvements in circuit parameters such as spatiotemporal resolution, reversibility, and orthogonality. Having outlined their characteristics, we next discuss cross‐modal integration, existing real‐life applications, translational hurdles, and emerging technological directions.

## 3 Real‐Life Applications of Traceless Genetic Circuitry

Despite their recent emergence, traceless gene circuits have already moved beyond proof‐of‐concept and demonstrated translational potential across many areas of medicine, as well as finding applications in industry and agriculture. In diabetes, opto‐, sono‐, and electrogenetic circuits have been used to remotely control insulin secretion via implanted cells to achieve seamless glycemic control in vivo [62, 70, 84, 97]. In neurological disorders, ultrasound‐based gene switches have shown promise in providing millisecond‐scale precision in neuromodulation and neuronal manipulation within an intact mammalian brain [119]. In cancer, light‐ and heat‐guided gene/photothermal combination therapies offer enhanced noninvasiveness and reduced side effects compared to chemotherapy and radiotherapy [120]. More recently, traceless switches have been incorporated in bacteria, to provide safe and precise regulation of local therapeutic payload expression in bacteria‐based therapies [121, 122]. Furthermore, engineered photoactivatable RNA base editors have achieved amelioration of clotting defects in patients with hemophilia B, opening the door to reversible and spatiotemporally specific modulation of base editing to target hereditary disorders [73]. In industrial biotechnology, traceless switches enable additive‐free modulation of fermentation and recombinant production of high‐value proteins [123, 124, 125]. Last but not least, in agriculture, optogenetic and thermogenetic switches afford transgene expression control as well as climate adaptation in crops [126, 127, 128, 129].

## 4 Discussion and Future Directions

The exponential growth of synthetic biology over the last few decades has opened up a vast number of opportunities in the fields of medicine, agriculture, sustainable energy, and high‐value chemical manufacturing [11, 12, 13, 14, 15]. Genetic switches form an indispensable part of synthetic genetic circuitry [130], and advancements in the molecular biology repertoire and the advent of artificial intelligence and machine learning have increasingly driven the development of new tools, including gene switches regulated by ‘traceless’ physical cues [131, 132, 133], extending the frontiers of synthetic biology [134]. Indeed, the integration and coupling of gene switches with biosensors detecting metabolites, hormones, or biomarkers is enabling the construction of next‐generation theranostic cell‐ and gene‐based therapies consisting of closed‐loop, autonomous systems that self‐regulate in response to deviations from the homeostatic state [130, 135, 136, 137]. Traceless gene switches inducible by physical cues, especially electrogenetic switches, are of particular interest due to their compatibility with the electronic world. Indeed, the rise of wearable electronics and bioelectronic implants is creating exciting opportunities for seamless bio‐digital interfaces [51, 61]. In particular, the dimmer switch‐like nature of traceless gene switches provides extremely high spatiotemporal resolution that is otherwise not possible in most biological systems, based on optimization of energy parameters like wavelength, frequency, amplitude, etc., depending on patient‐specific needs.

**FIGURE 7:**
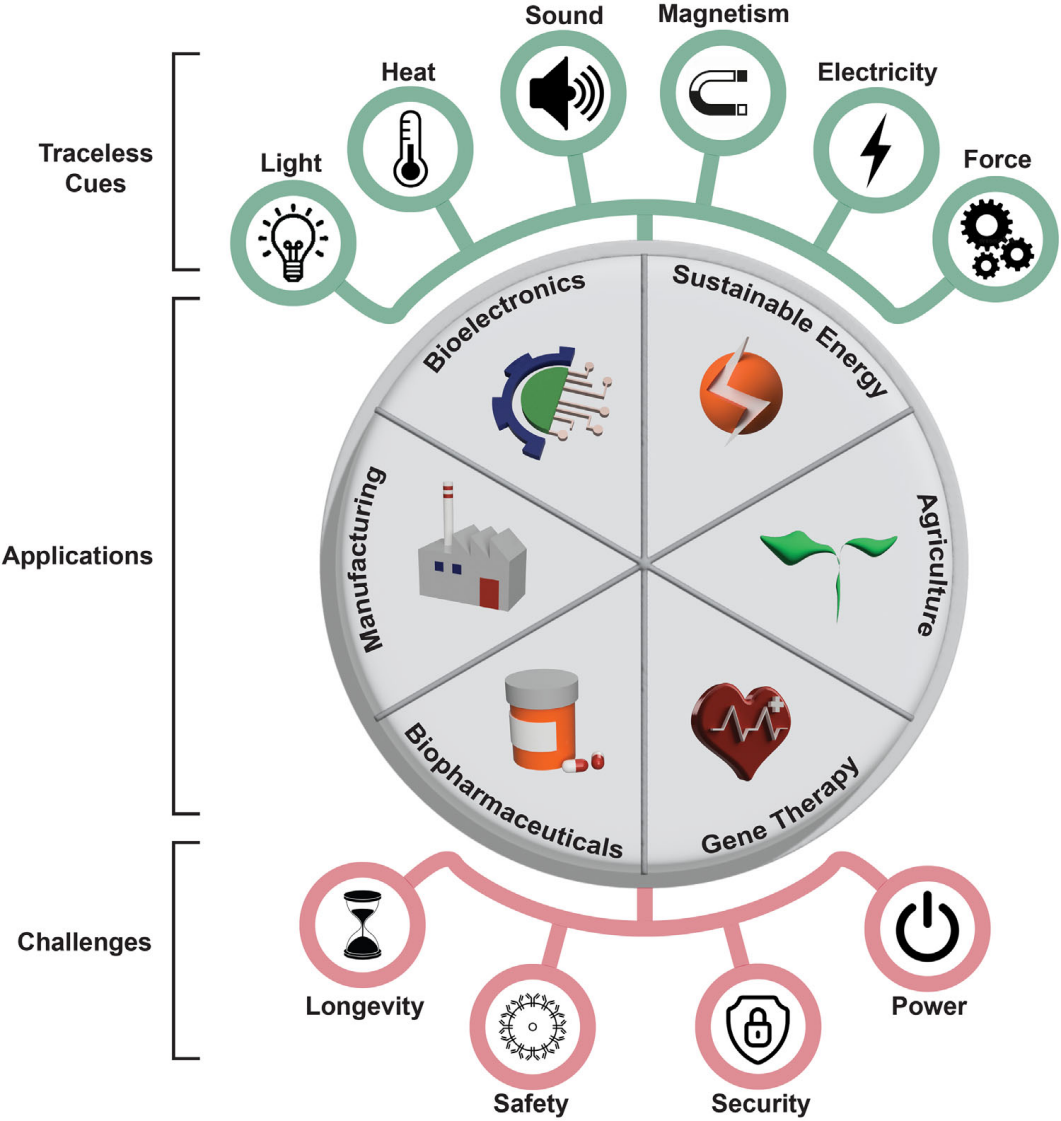
Traceless gene switches as universal control ports. Traceless gene switches have numerous trigger modalities within the energy spectrum, including light, heat, sound, magnetism, electricity, and mechanical force. Their applications already span gene therapies, biopharmaceutical production, industrial manufacturing, sustainable energy, wearable bioelectronics, and agriculture. With the continuing technological advancements in synthetic biology, traceless gene switches are likely to become key components of molecular biocomputers capable of Boolean logic. There remain important challenges in the areas of longevity, safety, security, and powering needs, but their resolution would firmly unite the computational power of biology with modern electronics.

A key issue in the regulation of synthetic genetic circuitry remains safety, and numerous hybrid and multimodal chemical switches have been developed using logic gates for enhanced control and safety [138, 139]. The synthetic multiplexing of orthogonal switches allows the use of Boolean logic operations in biological systems, and cells that incorporate such designer circuits can be considered truly ‘programmable’ [139]. The mixing of cellular computation with digital architecture enables bidirectional information exchange between biological processes and silicon‐based systems. Indeed, the integration of everyday consumer electronics like smartphones, smartwatches, and smart rings with therapeutic circuits enables geography‐independent control, where a patient in Europe can in principle fine‐tune a therapeutic implant while receiving algorithmic guidance from a physician in the United States [140]. Furthermore, the paradigm shift from chemical to physical cues greatly improves the capabilities of multimodal switches by removing pharmacokinetic constraints. Nevertheless, with the progress toward real‐life applications, questions regarding long‐term safety, user‐friendliness, and reversibility of gene switches still remain to be answered; in particular, fail‐safe operation will be critical [61]. Technical improvements are also required in various areas (Table 1). For instance, optogenetic and thermogenetic circuits suffer from poor deep‐tissue targeting; therefore, innovations in minimally invasive inducer delivery are needed to broaden clinical applicability. Similarly, the requirement for exogenous chromophores and nanoparticles for many optogenetic and thermogenetic circuits highlights the need for co‐factor‐free designs, particularly for magnetogenetics [109, 141]. Another issue is biocompatibility [106]. The functionality of implantable devices and engineered cell capsules can be degraded by fibrosis, immune rejection, and signal dampening over time [142]. The dependence on exogenous components also raises concerns about immune activation, accumulation, and clearance [143]. Additionally, the performance of electrogenetic implants can be impaired by electrode encapsulation and material degradation [103]. Addressing these challenges will require interdisciplinary development of novel biomaterials and long‐term in vivo studies to assess both device stability and circuit robustness [144, 145, 146].

**TABLE 1:** Comparative performance and key limitations of traceless gene switches.

Performance		Traceless	Cue		
parameter	Light	Heat	Sound	Electricity	Magnetism
Spatial Resolution	µm‐scale, focused beam lasers [65]	low due to heat diffusion [81]	mm‐scale focused ultrasound [86]	low (field) to moderate [97, 98]	low due to coarse field targeting [114]
Temporal Resolution	millisecond‐scale [65]	low due to heat diffusion [81]	millisecond‐scale [86]	millisecond‐scale [98]	seconds to minutes [147]
Dynamic Range / Fold‐change	>20–100x [148]	<20x [82]	<20x [84]	<20x [98]	<10x [114]
Reversibility	excellent [68]	good (cooling delay) [82]	excellent [84]	excellent [98]	variable due to nanoparticle aggregation [108]
Orthogonality / Crosstalk	moderate, ambient light triggers [66]	low [81]	moderate, ambient sound triggers [84]	moderate, ambient fields, nonspecific ROS formation [86]	high [149]
Multiplexability	wavelength‐based channels for logic control	limited	frequency‐based channels for logic control	limited	limited
Key limitations	poor tissue penetration, exogenous chromophores	temperature homeostasis	nonspecific activation due to ambient noise	short circuits	weaker response, exogenous nanoparticles

Although synthetic biology initiatives are often associated with preclinical and clinical applications, they also hold potential in non‐medical domains [11, 14]. Optogenetic and electrogenetic circuits have been used in manufacturing for precise regulation of fermentation and recombinant protein expression, serving to fine‐tune the industrial production of high‐value compounds [123, 124, 150, 151]. It is particularly appealing to capitalize on traceless cues in large‐scale industrial production, where minimizing the need for chemical additives is essential for regulatory compliance [15, 22, 26, 28]. In environmental science, traceless gene switches could allow wireless activation of pollutant detectors or remediation circuits [152, 153]. Plant synthetic biology already incorporates optoswitches to offer climate‐resilient crop regulation in agriculture [23, 154, 155]. Thus, the spectrum of potential applications for traceless genetic circuitry is vast (Figure 7).

Achieving the true translational potential of traceless switches across diverse applications hinges on resolving the current bottlenecks in the field. First, in clinical applications where parameters such as longevity, stability, and reliability are of utmost importance, the circuit‐organism interface needs to provide matrices capable of supporting the traceless induction in vivo without significant signal decay over extended periods of the order of months. Indeed, the aqueous, biochemically corrosive, immunoreactive environment in vivo calls for improvements and rigorous long‐term in vivo validation not only in gene delivery methods, but also in the biomaterials interfaces, which often still remain unvalidated beyond proof‐of‐concept studies [103, 105, 106]. Second, while traceless gene circuits offer notable advantages over chemically induced circuits, they remain open‐loop in the sense that they require active user input. In contrast, state‐of‐the‐art closed‐loop circuits can provide therapeutic effect autonomously [137, 156, 157]. To address this, electrogenetic sensor interfaces could be coupled with the traceless gene switches to automate the induction process, providing adaptive feedback algorithms to mitigate signal diffusion and off‐target activation. Lastly, while digitalization of the trigger signal offers unmatched spatiotemporal resolution, it introduces new limitations, the foremost being the necessity for a foolproof power source. Conventional batteries are not sustainable, alternating current is not portable, and renewable energy is not fully reliable. Pioneering efforts in in vivo power generation offer significant promise to tackle this challenge but are still far from what is needed for clinical translation due to their dependence on specific operating conditions [100, 101]. Furthermore, the digitalization of biological implants will inevitably grant them wireless capabilities, which will make it necessary to consider security against potential cyberattacks [158, 159]. Taken together, resolving these bottlenecks will require multidisciplinary collaboration from materials scientists, synthetic biologists, and engineers. Nevertheless, despite the challenges ahead, it seems likely that gene switches, particularly traceless ones, will gradually become universal biological control ports interfacing physiology with the electronic world [58, 59, 160]. Indeed, in the not‐so‐distant future, we may be able to say, “Hey Siri, adjust my blood glucose” or “Hey Google, fine‐tune my cholesterol levels.”

## Conflicts of Interest

The authors declare no conflict of interest.

## Data Availability

The authors have nothing to report.
